# Small Residues Inhibit Homo-Dimerization of the Human Carbonic Anhydrase XII Transmembrane Domain

**DOI:** 10.3390/membranes11070512

**Published:** 2021-07-07

**Authors:** Florian Cymer, Dirk Schneider

**Affiliations:** 1 Department of Chemistry, Biochemistry, Johannes Gutenberg University Mainz, 55128 Mainz, Germany; floriancymer@gmx.de; 2 Institute of Molecular Physiology, Johannes Gutenberg University Mainz, 55128 Mainz, Germany

**Keywords:** carbonic anhydrase XII, GxxxG, transmembrane domain, helix–helix interaction, small amino acids, interaction motif, interaction propensity, GALLEX

## Abstract

Amino acids with small side chains and motifs of small residues in a distance of four are rather abundant in human single-span transmembrane helices. While interaction of such helices appears to be common, the role of the small residues in mediating and/or stabilizing transmembrane helix oligomers remains mostly elusive. Yet, the mere existence of (small)xxx(small) motifs in transmembrane helices is frequently used to model dimeric TM helix structures. The single transmembrane helix of the human carbonic anhydrases XII contains a large number of amino acids with small side chains, and critical involvement of these small amino acids in dimerization of the transmembrane domain has been suggested. Using the GALLEX assay, we show here that the transmembrane domain indeed forms a strong transmembrane helix oligomer within a biological membrane. However, single or multiple mutations of small residue(s) to isoleucine almost always increased, rather than decreased, the interaction propensities. Reduction of helix flexibility and of protein–lipid contacts caused by a reduced lipid accessible surface area likely results in stabilization of helix–helix interactions within the membrane.

## 1. Introduction

While studied to a great extent in recent decades, the principles guiding folding of α-helical membrane proteins and proper assembly on individual α-helices are still only rudimentarily understood. The human GpA TM helix became a paradigm for studying sequence specificity in TM helix oligomerization. The sequence LIxxGVxxGVxxT drives the interaction of the GpA TM helix [1], and especially the motif of two Gly residues in a distance of four (the GxxxG-motif) has been identified to be key for dimerization [1,2,3]. In an in vivo screen selecting sequences driving strong homo-dimerization of a random library of TM helices, the GxxxG-motif also appeared [4], which indicated a more generalized importance of this motif in mediating TM helix interactions. In support of this, statistical analyses have shown that this motif is highly overrepresented in TM helices [5]. Further studies have indicated that other residues with small side-chains (Ala, Ser, Cys) can also mediate sequence-specific TM helix dimerization (discussed in [6,7]), and thus the motif is better described as “(small)xxx(small)”.

Small residues in a distance of four allow two TM helices to come into close contact enabling the formation of stabilizing Van der Waals interactions of neighboring residue side-chains [6,7,8,9]. Furthermore, a close distance positions hydrogen-bond donors, i.e., the side chains of Ser or Cys, in such a way that allows the formation of hydrogen bonds with backbone carbonyl oxygens of the interacting helix [10]. In addition, the formation of hydrogen bonds between the Gly Cα hydrogens and a backbone carbonyl oxygen of the interacting helix may contribute to the formation and stability of a TM helix dimer [11,12]. (Small)xxx(small) motifs typically stabilize right-handed helix dimer structures with an average crossing angle of about −40° [13]. This dimerization motif can be extended by other small residues to a (small)xxx(small)xx(small) “glycine zipper” motif [14]. These intriguing observations have prompted many researchers in the past to search for such motifs in their TM helix of interest, and the mere existence of such a motif was often used as a predictor for TM helix interactions. Yet, not solely the motif but also the sequence context is crucial for (small)xxxx(small)-mediated TM helix dimerization, which clearly is important for ensuring the formation of proper TM helix dimers in cellular membranes where multiple TM helices containing such motifs are present at a time. Yet, while two interacting helices obviously have to have complementary surfaces, interaction might not be pinned down to defined motifs consisting of small residues. In fact, the stability of a GxxxG-mediated TM helix dimer is affected by surrounding residues [15,16,17,18], and the involvement of other residues and motifs in sequence-specific dimerization of TM helices has been described in past decades [19,20,21,22,23,24,25,26]. Yet, small residues are rather abundant in human TM helices, especially in single-span TM helices, which even contain on average at least one canonical GxxxG motif [7,27,28,29,30]. While the interaction of several single-span TM helices has been analyzed to some extent and appears to be common, the role of the (small)xxx(small) motifs and/or of small residues in mediating and/or stabilizing TM helix dimers remains mostly elusive.

In the present study, we have used the human carbonic anhydrase (CA) XII TM helix as a model to systematically study the impact of replacing small residues in a TM domain on the in vivo interaction propensity of the TM segment. First, we show that the CA XII TM domain does indeed have an intrinsic interaction propensity in vivo. Interaction of the wt and mutated CA XII TM segment was analyzed using the GALLEX-system, a bacterial two-hybrid system, allowing the analysis of TM helix–helix interactions within a biological membrane [31,32]. While the CA XII TM domain forms a strong TM helix dimer in a biological membrane, single or multiple mutations of small residue(s) to the β-branched amino acid Ile almost always led to increased interaction propensities. Thus, small residues in the CA XII TM helix block, rather than mediate, TM helix dimerization within a biological membrane. As discussed, this could be due to changes in helix dynamics and/or a decrease of the lipid accessible surface area. Our study highlights that TM helix dimerization and the structure of TM helix dimers should be predicted with caution when mainly based on the mere existence of (small)xxx(small) motifs within the TM domain.

## 2. Materials and Methods

### 2.1. Plasmid Construction

Synthetic oligonucleotide cassettes coding for the human CA XII TM domain were ligated into a *Nhe*I and *BamH*I restriction-digested pBLM plasmid [32], resulting in the plasmid pBLMCAXIITM. Correct ligation was confirmed by DNA sequencing. Mutations were introduced via the Quick Change site-directed mutagenesis kit (Agilent, Santa Clara, CA, USA) following the supplier’s instructions using the pBLMCAXIITM plasmid as a template. Mutations were confirmed by sequencing.

### 2.2. GALLEX Assay 

The GALLEX system was used to measure CA XII TM helix oligomerization as described in detail elsewhere [31,32]. Briefly, the pBLMCAXIITM construct and variants were used to transform *E. coli* SU101 cells. Addition of 100 µM IPTG resulted in the expression of a chimeric protein consisting of an N-terminal LexA DNA-binding domain fused to the CA XII TM domain and the maltose-binding protein at the C-terminus. As a mock control, we measured the empty pBLM plasmid in the GALLEX system. As a positive control in the GALLEX assay, dimerization of the well-studied and strongly interacting glycophorin A (GpA) TM domain was analyzed. As a negative control, the weakly interacting G83I GpA-variant was assayed.

Proper membrane insertion was tested for all expressed chimeric proteins by extracting soluble and membrane-attached proteins from isolated *E. coli* membranes with 0.1 M NaOH. Only proteins integrated within the membrane remain in the membrane after this treatment. Membrane-integrated chimeric proteins were detected via Western blot analysis using a maltose-binding protein-specific antibody (New England Biolabs, Frankfurt, Germany).

Proper transmembrane orientation of the chimeric proteins was tested by plating the maltose-binding protein-deficient *E. coli* strain NT326 [33], transformed with the pBLMCAXIITM and control plasmids, on M9 minimal media agar plates. The plates contained IPTG to induce recombinant protein expression, 100 µg/ml ampicillin as a selection marker, and maltose as the only carbon source. Bacterial growth can only occur if the maltose-binding protein is located within the periplasm and thus the chimeric protein is inserted in the correct topology.

### 2.3. Software

A model of the human CA XII TM helix dimer was created using the PREDDIMER server [34]. Sequences were aligned using the T-COFFEE algorithm [35] and carbonic anhydrase sequences of different mammalian species were derived from the ExPASy proteomic server [36]. The lipid accessible surface area was calculated with the GETAREA program [37] using the previously generated CA XII TM domain model and energy-minimized models of CA XII TM domain variants. In order to probe the lipid accessible surface area, a probe radius of 1.88 Å was chosen, which corresponds to the radius of a methyl group and has been used for this purpose previously [38].

## 3. Results and Discussion

The mere existence of a (small)xxx(small) motif is often used to predict a dimeric TM helix structure. For example, the structures of the soluble domains of the human CA IX and XII have been solved in recent years, and the structures of the TM regions have been modeled [39,40]. In tumors, these isoenzymes are overexpressed and contribute to an elevated tumor progression by acidifying the surrounding extracellular space, leading to elevated tissue penetration [41,42]. The human CA XII is a bitopic membrane protein located in the plasma membrane of different tissues. While the crystal structure of the CA XII extracellular domain has been solved [39], the structures of the short intracellular domain-containing putative phosphorylation sites and of the TM domain are still elusive. As the catalytically active domains are dimeric in solution, and the TM domains are rich in small residues and contain (small)xxx(small) motifs, a dimeric TM helix structure was assumed and modeled using the GpA dimer structure as a template [39], however, without experimental proof. Actually, it is currently still unknown whether the CA XII TM domain dimerizes at all in a biological membrane.

**Figure 1:**
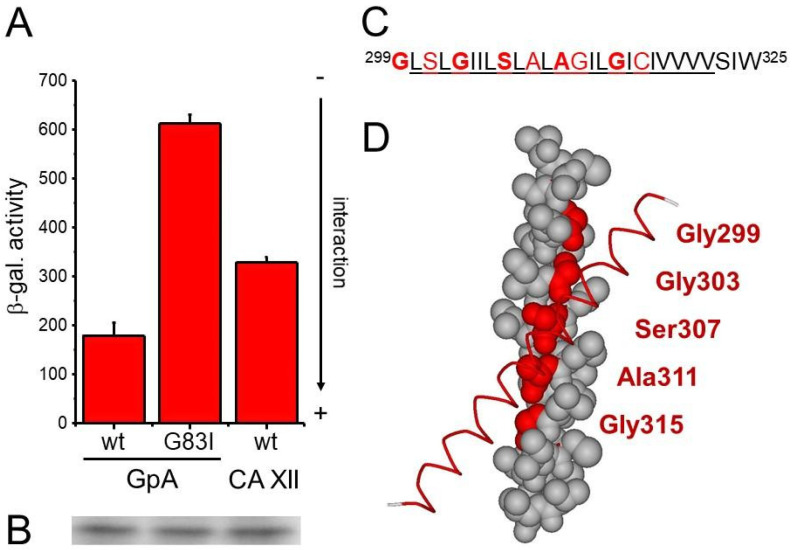
Human CA XII TM helix–helix interaction. (**A**) When measured with the GALLEX assay, the wild-type CA XII TM domain has a significant interaction propensity compared to the strongly interacting TM domain of human GpA and the weakly interacting GpA variant G83I. The interaction propensities are an average of three independent measurements and the standard deviation is shown. (**B**) Identical amounts of isolated total membrane proteins of cells analyzed in (**A**) were separated in an SDS gel, and the expressed amount of the Ca XII TM fusion protein was tested via Western blot analyses using an antibody directed against the MalE protein. All analyzed proteins were expressed at similar levels. (**C**) Sequence of the human CA XII TM region. The TM regions as predicted by TMHMM are underlined. Small residues are highlighted in red. Small residues at a distance of four are highlighted in bold. (**D**) Model of the human CA XII TM domain dimer predicted by the PREDDIMER server. CPK representation of one TM domain with highlighted and labeled small residues Gly299, Gly303, Ser307, Gly311, and Gly315. The interacting helix is shown as a Cα wire.

More than 40% of the amino acids within the predicted human CA XII TM domain have small side-chains (Figure 1C). The overall sequences of mammalian CA XII TM domains are highly homologous, and small residues are well-conserved. The high abundance of small amino acids potentially enables close packing of TM helices.

To study the CA XII TM helix interaction propensity in a biological membrane in detail, we performed GALLEX measurements. The GALLEX system allows measuring the interaction propensity of TM helices in vivo, within the *E. coli* inner membrane [32]. In the GALLEX system, a TM helix of interest (here, the CA XII TM helix) is genetically fused at its N-terminus to the DNA-binding domain of the *E. coli* LexA protein and at its C-terminus to the *E. coli* MalE protein, which facilitates membrane insertion. Only a homo-dimeric LexA DNA-binding domain can bind to a promoter/operator region located within the *E. coli* genome and thereby repress the activity of the *lacZ* reporter gene (coding for β-galactosidase). As dimerization of the DNA-binding domain is mediated by interactions of the fused TM domains, the determined activity of the reporter directly correlates with a given interaction propensity. Besides studying the interactions of CA XII TM helices, as controls, we also determined the interaction propensity of the well-studied human GpA TM helix, which forms a highly stable TM helix dimer, as well as a GpA G83I variant, which interacts rather weakly due to the mutation of a Gly residue, which is critically involved in TM helix dimerization [1,2,3]. Noteworthily, while measured in a bacterial membrane, GALLEX and related genetic systems, such as ToxR, TOXCAT, and POSSYCCAT (or variations of these), have frequently been used in the past to study interactions of human TM proteins, e.g., in [3,10,15,16,17,18,25,28,29,43]. Nevertheless, while differences in a given interaction propensity caused by a mutation will likely be observed in a non-native, yet biological, membrane environment, the actual interaction strength might vary depending on the exact membrane lipid composition [6].

The interaction propensity of the wt CA XII TM domain is less pronounced than the interaction propensity of the strongly interacting GpA TM helix, yet significantly stronger than the weakly interacting GpA G83I variant (Figure 1A). Thus, these data demonstrate that the human CA XII TM domain has an intrinsic propensity to self-interact within a biological membrane, which most likely contributes to the stability of the dimeric full-length protein.

Putative structures of the human CA XII TM helix dimer have been predicted recently using the program CATM [44]. Three prominent structures have been modeled, which all have a crossing of −47° to −48°. In the top-scoring model, the residues Ala311 and Gly315 are at the helix crossing point and thus are predicted to be key for dimerization. While Models 2 and 3 differ slightly, in both models, the crossing point of the TM helices mainly involves the residues Gly299 and Gly303. When the structure of the putative CA XII TM helix dimer was modeled using the program PREDDIMER [34], the top-scoring model also predicted a right-handed helix dimer with a crossing angle of −45°. Yet, interaction of the helices is mainly mediated/stabilized by the residues Gly303 and Ser307 (Figure 1D). Further dimeric (left- as well as right-handed) structures were also predicted, where other small residues are involved in helix–helix interactions. However, essentially none of the models agreed with the CATM predictions. With THOIPA (Transmembrane Homodimer Interface Prediction Algorithm), which predicts interfacial residues from evolutionary sequence data [45], the residues Gly299 and Gly303 are predicted to lay at the helix–helix interface, in line with Models 2 and 3 predicted by CATM.

Actually, the small residues Gly299, Gly303, Ser307, Ala311, and Gly315 (highlighted in Figure 1D) form a continuous groove along the CA XII TM helix. Packing of the bulkier residues Leu302, Leu306, Leu310, and Leu314, which form a ridge on the adjacent helix, into this groove might allow close packing of two helices, and diverse small residues might be involved in the formation of different CA XII TM helix dimer structures, which might be involved in regulation of the CA XII activity.

**Figure 2:**
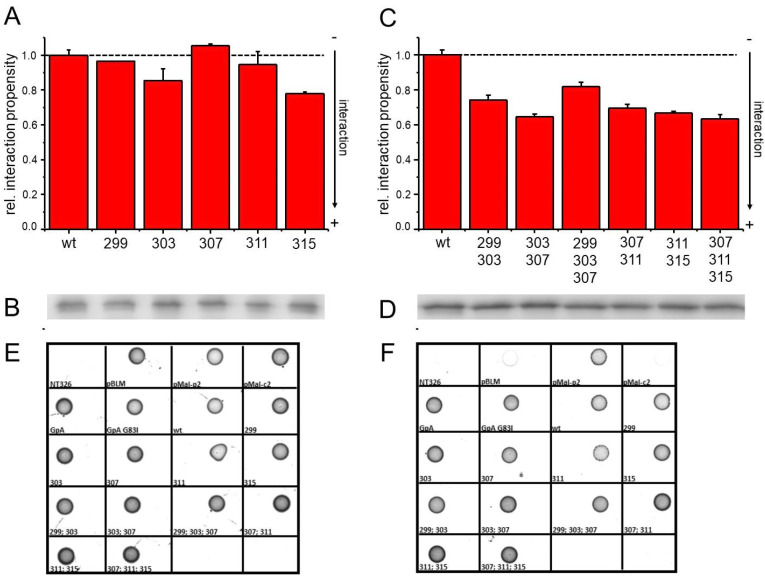
GALLEX measurements of the human CA XII TM domain mutants. (**A**) Interaction tendencies of CA XII TM variants carrying mutations of individual small amino acids to Ile. The position of the mutated amino acid is indicated below each bar. All interaction propensities have been normalized to the wt (set as 1.0). The interaction propensities are an average of three independent measurements, and the standard deviation is shown. (**B**) Identical amounts of isolated total membrane proteins of cells analyzed in (**A**) were separated via SDS-PAGE, and the expressed amount of the CA XII TM fusion protein was analyzed via Western blot analyses using an antibody directed against the MalE protein. All analyzed proteins were expressed at similar levels in membranes. (**C**) GALLEX measurement of CA XII variants carrying mutations of multiple small amino acids to Ile. All interaction propensities have been normalized to the wt (set as 1.0). The interaction propensities are an average of three independent measurements, and the standard deviation is shown. (**D**) Identical amounts of isolated total membrane proteins of cells analyzed in (**C**) were separated in an SDS gel, and the expressed amount of the Ca XII TM fusion protein was analyzed via Western blot analyses using an antibody directed against the MalE protein. All analyzed proteins were expressed at similar levels in membranes. (**E**, **F**) Maltose complementation assay to determine the TM topology of the analyzed chimeric proteins. The correct topology of the analyzed chimeric proteins within the *E. coli* membrane was tested using a maltose complementation assay. MalE-deficient *E. coli* NT326 cells carrying different plasmids are able to grow on M9-agar plates containing glucose as the sole carbon source (**E**), but only grow on maltose plates (**F**) if the construct is correctly oriented in the membrane and the MalE domain is located in the *E. coli* periplasm. The plates contained 100 µg/ml ampicillin and a final concentration of 100 µM IPTG. On both plates, growth of NT326 cells, which have not been transformed with a plasmid, is not seen. Cells containing the empty pBLM plasmid and cells carrying the pMal-c2 plasmid (which leads to a cytoplasmic localization of the MalE domain) are unable to grow on maltose plates. Growth of all used GALLEX constructs on maltose-only plates confirms membrane insertion and a correct TM orientation of the CA XII TM domains.

In order to further elucidate the function of the small residues forming the continuous groove in the TM domain, we consecutively mutated the small residues highlighted in Figure 2B to Ile. We anticipated that the replacement of small amino acids by the bulkier Ile residue would disturb close packing interactions and thereby disrupt TM helix/helix interactions, a strategy used several times before (e.g., [1,43,46]. Surprisingly, all mutations of the selected small residues to Ile led to either no changes or to slightly increased interaction propensities (Figure 2A), indicating that the voids or grooves formed by the small residues are eventually not directly involved in TM helix dimerization. To further analyze this, we subsequently mutated multiple small amino acids within the CA XII TM domain in parallel and monitored the interaction tendencies of the variants. As shown in Figure 2C, the interaction tendencies of the double and even triple mutants did not decrease at all, yet a significant increase in the interaction propensities was observed for all mutated TM sequences. Simultaneous mutations of the small amino acids at positions 299/303, 303/307, 307/311, or 311/315 resulted in a stronger interaction compared to the wt or the single mutated TM helices (Figure 2C). Introducing three mutations in parallel at positions 307/311/315 even resulted in an additional increase in the interaction propensity. Solely the triple mutant 299/303/307 displayed a slightly reduced interaction propensity in comparison to the respective double mutants. However, this triple mutant still had a stronger interaction propensity than the wt TM helix. Together, these results clearly demonstrate that, while the CA XII TM helix has an intrinsic propensity to self-interact, the small amino acids appear to destabilize rather than stabilize an oligomeric TM structure. Noteworthily, the expression level was controlled for all constructs, and the concentration of the individual proteins within the *E. coli* inner membrane was similar (Figure 1B and Figure 2B,D). Furthermore, all proteins had the expected TM topology, with the TM helix C-terminus facing the bacterial periplasm (Figure 2E,F).

The unexpected results of our measurements forced us to reevaluate our initial assumption that the replacement of a small residue by Ile typically disrupts helix–helix interactions. The presented observations indicate that the mutated residues are not a critical part of a well-defined interaction interface, as their mutation does not hinder, but rather increases, the interaction propensity of the CA XII TM domain in most cases. GxxxG and GxxxG-like motifs are well-known to frequently mediate helix–helix interactions, but it is also becoming increasingly apparent that there are a host of unknown modes of helix–helix interactions (for a review, view [6,7,47]).

The TM domain of the human CA XII consists of more than 40% of amino acids with small side chains, leading to the formation of a rather flexible TM helix structure. In fact, as for Gly-containing TM helices, it has been shown that Gly increases the flexibility of a given TM helix [48] and thus weakens the regular “stiff” helix geometry. In TM helices, the helical propensity of an amino acid correlates with its side-chain hydrophobicity [49]. Replacing small residues with a low helical propensity in membranes by Ile, a residue that severely promotes the formation of ideal TM α-helices [49], likely reduces the flexibility and structural dynamics of the CA XII TM helix, shifting the monomer–dimer equilibrium towards the dimer. Furthermore, intra-helical interactions between β-branched amino acids might additionally contribute to the overall stability of a TM helix [5,49]. Since the increase in the interaction propensity caused by the introduction of β-branched residues seems to depend on the distance to other such residues, the increase in interaction propensity of single Ile mutants should also depend on the sequence context with respect to the presence of other β-branched side chains. Indeed, the interaction propensity of the CA XII variant having a single mutation to Ile at position 303 is slightly increased. Interestingly, in the immediate vicinity of residue 303, two Ile are located at positions 304 and 305, and Ile303 might interact with the neighboring side chains. A far more pronounced increase in the interaction propensity was observed when Ile was introduced at position 315, and in the distance of four, a Val residue is located at position 319. Intrahelical interactions of β-branched side chains are strongest in i, i + 4 and weaker in i, i + 3 [50]. The multiple mutations to Ile always led to a positioning of Ile in the pattern i, i + 4. Here, a strong increase in the interaction propensity was always observed, whereas single mutations to Ile at these positions are frequently without effect. Noteworthily, in the present study, exclusively mutations to Ile were analyzed, and it is feasible to assume interactions with other β-branched side chains. Consequently, mutations to other amino acids can clearly have a different effect on the observed interaction propensities. 

**Figure 3:**
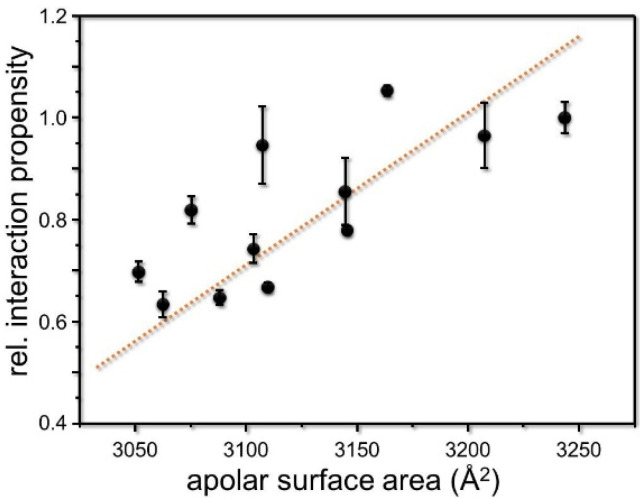
The lipid accessible apolar surface area correlates with the normalized interaction propensity of the wild-type CA XII TM segment and its variants. A linear fit of the correlation is shown. (r^2^ = 0.75. The normalized standard deviations of the three independent measurements from the GALLEX experiments are indicated. The lipid accessible apolar surface area was calculated using the GETAREA program [37] and energy-minimized modeled structures of the respective CA XII TM helix variant.

In the case of the well-studied GpA TM helix dimer, the interaction propensity of the (mutated) GpA TM helix was inversely related to the interfacial lipid accessibility of the GpA helix dimer [38]. Thus, we also analyzed a potential correlation of the apolar lipid accessible surface area of the analyzed CA XII TM segments and the measured interaction propensities. Interactions between CA XII TM helix side-chains and lipids might stabilize the structure of the monomeric TM helix, thereby inhibiting the formation of TM helix dimers. In fact, a correlation between the lipid accessible surface area of the TM helices analyzed in this study and the observed interaction propensity was found (Figure 3). Successive introduction of Ile residues resulted in a less lipid-solvated TM helix, promoting helix–helix interactions. 

## 4. Conclusions

Our mutational analyses suggest that the numerous small amino acids are not critically involved in the dimerization of the CA XII TM helix, but rather destabilize the oligomeric structure. Small amino acid side chains might enhance the flexibility of the CA XII TM helix backbone, which has an impact on the lipid shell surrounding the helix. Introduction of Ile residues results in a less flexible TM helix structure, which is less lipid solvated, and thus reduction of protein–lipid contacts and of helix flexibility likely promotes interactions of the CA XII TM helix. Future studies to analyze the impact of small residues and small-xxx-small motives, especially with single-span human TM helices that contain multiple small residues and even contain on average at least one canonical GxxxG motif [7,27,28,29,30], will further enhance our knowledge of the role of small residues in TM helix association. Nevertheless, the mere existence and evolutionary conservation of (multiple) small residues and/or of (small)xxx(small) motifs in TM helices is probably not a good predictor for assuming GpA-like TM helix–helix interactions, and structural modeling using such motifs as the key predictor likely falls too short.

## Data Availability

The data that support the findings of this study are available on re-quest.
